# Membrane Compartmentalization and Scaffold Proteins in Leukocyte Migration

**DOI:** 10.3389/fcell.2020.00285

**Published:** 2020-04-28

**Authors:** Guerric P. B. Samson, Daniel F. Legler

**Affiliations:** ^1^Biotechnology Institute Thurgau at the University of Konstanz, Kreuzlingen, Switzerland; ^2^Faculty of Biology, University of Konstanz, Konstanz, Germany; ^3^Theodor Kocher Institute, University of Bern, Bern, Switzerland

**Keywords:** leukocyte migration, membrane compartmentalization, scaffold proteins, flotillin/reggie, tetraspanin, caveolin

## Abstract

Leukocyte migration across vessels into and within peripheral and lymphoid tissues is essential for host defense against invading pathogens. Leukocytes are specialized in sensing a variety of guidance cues and to integrate environmental stimuli to navigate in a timely and spatially controlled manner. These extracellular signals must be transmitted across the leukocyte’s plasma membrane in a way that intracellular signaling cascades enable directional cell movement. Therefore, the composition of the membrane in concert with proteins that influence the compartmentalization of the plasma membrane or contribute to delineate intracellular signaling molecules are key in controlling leukocyte navigation. This becomes evident by the fact that mislocalization of membrane proteins is known to deleteriously affect cellular functions that may cause diseases. In this review we summarize recent advances made in the understanding of how membrane cholesterol levels modulate chemokine receptor signaling and hence leukocyte trafficking. Moreover, we provide an overview on the role of membrane scaffold proteins, particularly tetraspanins, flotillins/reggies, and caveolins in controlling leukocyte migration both *in vitro* and *in vivo*.

## Introduction

**FIGURE 1:**
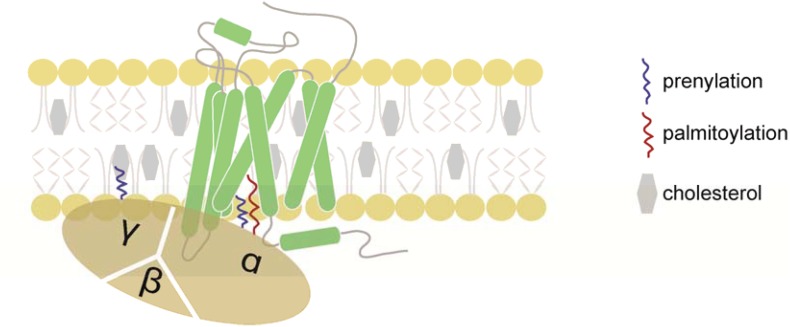
Schematic representation of a chemokine receptor and its associated heterotrimeric G-protein. Chemokine receptors belong to the GPCR family and possess seven-transmembrane domains. Chemokines initiate chemokine receptor activation by binding to the N-terminus and extracellular loops of the receptor. Once the chemokine is tethered to the receptor, the N-terminus enters the binding pocket where it interacts with the transmembrane domains of the chemokine receptor. The presence of cholesterol is critical for the stability of the chemokine receptor. Upon ligand binding, the receptor promotes the exchange of GDP for GTP on the Gα-subunit, resulting in the dissociation of the Gα- from the Gβγ-subunits and downstream signaling. The Gα- and Gγ-subunits are post-transcriptionally lipidated facilitating their association with the plasma membrane.

Cell migration is essential for a number of physiological and pathophysiological processes, such as embryogenesis, organogenesis, tissue homeostasis, but also cancer malignancy. In host defense, guided cell locomotion and positioning critically contributes to wound healing and cellular immune responses. Leukocytes are professional migratory cells that are able to sense various guidance cues and to integrate external signals to navigate through different types of tissue and to cross blood and lymph vessels (Nourshargh et al., 2010). Important guidance cues are provided by the chemokine network. Locally produced chemokines can form gradients *in situ* that migrating cells can sense through cognate chemokine receptors (Hughes and Nibbs, 2018). Chemokine receptors belong to the class A of G-protein coupled receptors (GPCRs) and possess seven α-helical domains that span the plasma membrane and are connected by extracellular and intracellular loops (Legler and Thelen, 2018; Lämmermann and Kastenmüller, 2019). Chemokine binding to the receptor induces conformational changes that markedly rearrange the positions of the transmembrane helices particularly at the cytoplasmic surface of the plasma membrane allowing G-protein coupling and signal transduction (Legler and Thelen, 2018; Weis and Kobilka, 2018). Chemokine receptors couple to heterotrimeric G-proteins of the G_*i*_ class and their activation promotes the exchange of GTP for GDP on the Gα-subunit resulting in its dissociation from the βγ-subunits (Figure 1). Notably, members of the small GTPase family transmit downstream signals and thereby link chemokine receptor activation to actin cytoskeleton rearrangements required for the induction of cell polarity and locomotion. Members of the Rho family GTPases, namely Rac1 (Benvenuti et al., 2004), RhoA (Pertz et al., 2006), and Cdc42 (Lämmermann et al., 2009), translocate to the plasma membrane upon activation (Collins, 2003). In general, Rac1 is known to control actin polymerization at the leading edge, while RhoA regulates myosin contraction at the rear of a migrating cell (Pertz et al., 2006; MacHacek et al., 2009).

**FIGURE 2:**
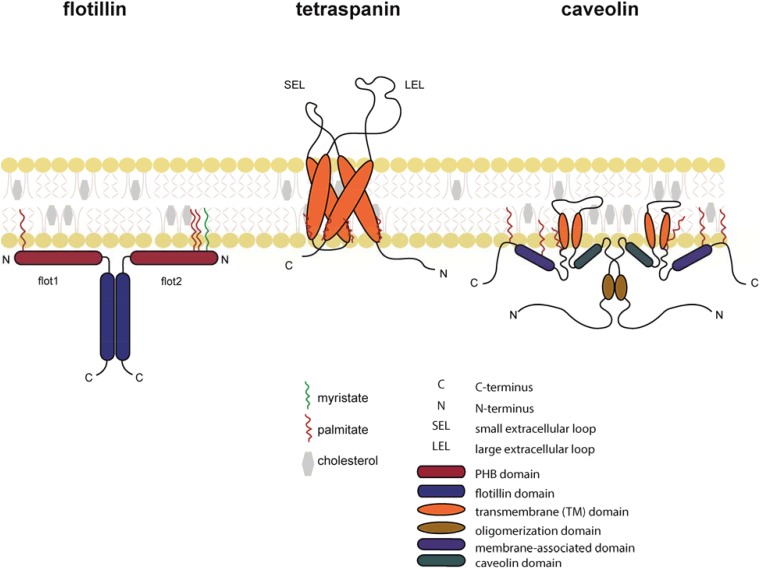
Schematic representation of flotillin, tetraspanin, and caveolin in the lipid bilayer. Flotillins are associated at the cytosolic leaflet of the plasma membrane through its N-terminal PHB domain. Membrane association is further assured through myristoylation (green) and palmitoylation (red). Flotillins form hetero-mers through specialized intracellular flotillin domains. Tetraspanins are composed of four transmembrane α-helices and two extracellular domains: the SEL (small extracellular loop) and the LEL (large extracellular loop). Tetraspanins are palmitoylated at a conserved CXXC motif in their transmembrane domains. Caveolins form hairpin loops that are inserted into the plasma membrane. Both N- and C-termini face the cytoplasmic side of the membrane.

As guided cell migration depends on extracellular signals that must be transmitted across the plasma membrane, it became obvious that the organization of the plasma membrane and membrane compartmentalization influence the cell’s ability to sense extracellular cues and to migrate. One of the most prominent concept for membrane compartmentalization refers to as the “lipid raft” hypothesis first described in 1988 (Simons and Van Meers, 1988) proposing that specialized subcompartments or microdomains of the lipid bilayer of the membrane control different cellular functions such as receptor endocytosis and signaling (Simons and Ikonen, 1997). In the 1990s, different membrane residing scaffold protein families were discovered, that affect the composition of the membrane (Figure 2). Proteins of the tetraspanin family integrate into the membrane through four transmembrane domains, whereas the flotillin/reggie family represent small cytoplasmic proteins that are hooked to the membrane by means of fatty acid oxidation (Seigneuret et al., 2001; Ficht et al., 2019). Finally, proteins of the caveolin (cav) family penetrate from the cytoplasmic site into the membrane through a hairpin-like structure and are further anchored into the membrane through palmitoylation/myristoylation (Dietzen et al., 1995; Figure 2). Briefly, tetraspanins have the ability to interact with other members of their family or with partner proteins such as integrins, adhesion molecules or signaling receptors to form “tetraspanin enriched microdomains” or “TEMs” (Hemler, 2005). The flotillin/reggie family consists of two members, flotillin-1 (flot1), also known as reggie-2, and flotillin-2 (flot2)/reggie-1 (Bickel et al., 1997; Schulte et al., 1997). Flotillins are known to hetero-dimerize and to assemble into larger complexes to act as scaffold (Langhorst et al., 2007; Neumann-Giesen et al., 2007). For example, in T cells, flotillins were shown to pre-assembly in caps to stabilize the immunological synapse and to as scaffold for the T cell receptor (TCR) machinery (Slaughter et al., 2003; Langhorst et al., 2006; Compeer et al., 2018). Members of the cav family are best known for the formation of cave-like membrane structures termed caveolae, membrane invaginations involved in endocytosis and signaling (Lefkir et al., 2003; Collins et al., 2012; Sotgia et al., 2012; Wang et al., 2015). In this review, we summarize the current understanding how cholesterol modulates chemokine receptor signaling and how membrane scaffold proteins regulate leukocyte migration.

## Membrane Microdomains, Lipid Rafts and Cholesterol

Amphiphilic phospholipids represent the major building block of lipid bilayers of vertebrate membranes. Phospholipids are composed of a hydrophilic phosphate head and two hydrophobic fatty acid tails, which vary in length and saturation and thereby account for the broad range of phospholipid species (Simons and Toomre, 2000). The fatty acyl groups of the phospholipids influence the membrane fluidity and hence the lateral mobility of membrane associated proteins (Krapf, 2018). In addition, cell membranes also contain the sterol cholesterol. Cholesterol molecules preferentially interact with saturated fatty acyl groups of phospholipids and thereby shift the membrane structure from a heterogeneous fluid membrane with high mobility to a more rigid and stiff membrane with lipid and protein patches (Legler et al., 2017). The original concept of “lipid raft” or “membrane microdomains” (Simons and Ikonen, 1997) has been further developed and refined. Although direct microscopic visualization of lipids rafts at millisecond rates still remains challenging (Klymchenko and Kreder, 2014; Sezgin et al., 2017; Kinoshita et al., 2018), recent new biophysical techniques confirmed the presence of such domains in cells and provided new insights in to the cell membrane heterogeneity (Sezgin et al., 2017). In addition, studies on crystal structures of proteins clearly revealed that cholesterol molecules can directly interact with membrane associated scaffold proteins. Notably, solving the crystal structure of the tetraspanin CD81 revealed a cholesterol-binding pocket at the cavity between the four transmembrane helices situated at the inner leaflet of the membrane (Zimmerman et al., 2016). Importantly, the presence of cholesterol within the cavity keeps CD81 in a closed conformation. Molecular dynamics analysis revealed that cholesterol dissociation from the binding pocked results in an open conformation of CD81 that facilitates a tetraspanin-dependent transport of CD19 to the cell surface (Zimmerman et al., 2016). These findings are in line with an earlier study showing that membrane cholesterol contributes to the organization of tetraspanin microdomains (Charrin et al., 2003). More generally, this property of cholesterol to modulate the mode of action of tetraspanins not only affects protein transport [e.g., CD81:CD19 (Zimmerman et al., 2016); CD9:MHCII (Silvie et al., 2003; Rocha-Perugini et al., 2009; Banse et al., 2018)], but also malaria or cytomegalovirus infection [through CD81 (Silvie et al., 2003; Rocha-Perugini et al., 2009; Banse et al., 2018)], and cell migration as described later. Similarly, early electron microscopy studies identified an important role of cholesterol for the assembly of caveolae (Rothberg et al., 1992) whose major constituent, caveolin-1, contributes to dendritic cell migration as discussed in a subsequent paragraph.

## Role of Cholesterol in Chemokine Receptor Crystallization, Signaling and Leukocyte Migration

Chemokine receptor activation is initiated by the binding of the chemokine to the extracellular N-terminus and extracellular loops of the receptors (Figure 1). Once the chemokine is tethered to its cognate receptor, its unstructured N-terminus is capable to enter the binding pocket where it interacts with the transmembrane bundles of the receptor. This leads to the rearrangement of the seven transmembrane helices of the receptor resulting in profound conformational changes across the plasma membrane (Kufareva et al., 2017; Legler and Thelen, 2018). Attempts to crystalize chemokine receptors, and GPCRs in general, revealed that addition of cholesterol is necessary to stabilize the receptor during the solubilization, purification, and crystallization processes (Wu et al., 2010; Qin et al., 2015). Evidence for a direct physical interaction between cholesterol and a GPCR has first been noted in the crystal structure of the β_2_-adrenergic receptor, where two cholesterol molecules were found to directly interact with a receptor monomer (Cherezov et al., 2007). Although chemokine receptors do not possess a consensus cholesterol binding motifs as many other GPCRs (Hanson et al., 2008; Thelen and Legler, 2018), chemokine receptors (i.e., CXCR4, CCR2, CCR5, and CCR7) had to be reconstituted into lipidic cubic phases containing at least 10% cholesterol prior to successful crystallization (Wu et al., 2010; Qin et al., 2015; Zheng et al., 2016, 2017; Jaeger et al., 2019). Importantly, cholesterol inclusion was shown to increase chemokine binding to solubilized CXCR4 (Babcock et al., 2003; Palmesino et al., 2016). By contrast, cholesterol depletion in cells reversibly inhibited ligand binding to the chemokine receptor CCR5 and resulted in attenuated signal transduction and cell migration (Mañes et al., 2000; Signoret et al., 2005). A regulatory role for cholesterol in chemokine receptor function derive from the discovery that CCR5 and CXCR4 serve as co-receptors for human immunodeficiency virus (HIV) infection and that cholesterol is essential for the budding and fusion of the virus envelope with the host plasma membrane (Hug et al., 2000; Simons and Ehehalt, 2002). In fact, the HIV glycoprotein gp120 binds to CXCR4 and CCR5 in cholesterol-enriched domains of the host cell (Mañes et al., 2000; Ono and Freed, 2001). The notion that changes in cholesterol levels in dendritic cells regulate their migratory capacity (Hauser et al., 2016) has gained significant attention. In fact, exposing dendritic cells to danger signals led to a marked downregulation of key enzymes involved in cholesterol biosynthesis, while proteins controlling cholesterol efflux were upregulated. Simultaneously, theses danger signals were shown to provoke oligomerization of the chemokine receptor CCR7 resulting in a pro-migratory dendritic cell phenotype (Hauser et al., 2016). Moderately modulating cholesterol levels using cholesterol lowering drugs not only affected CCR7 oligomerization, but also chemokine-driven migration (Hauser et al., 2016). By contrast, a complete depletion of cellular cholesterol interfered with the stability of the receptor manifested by impaired chemokine binding to the receptor and hampered chemotactic cell behavior (Nguyen and Taub, 2002). Molecularly, cholesterol-dependent CCR7 oligomerization enabled the activation of an additional oligomer-dependent Src kinase signal transduction pathway aside the classical G-protein-dependent signaling pathway. This conjoint signaling is possible as in a CCR7 dimer (or tetramer) scenario, one (or two) receptor-mer(s) are able to couple to the heterotrimeric G-protein, while the other (two) receptor-mer(s) interact with the Src kinase. Notably, Src is able to pre-associate with oligomeric CCR7, which upon chemokine activation, phosphorylates the receptor and creates a docking site for SH2-domain-bearing signaling molecules (Hauser et al., 2016). It is interesting to note that the α- and γ-subunits of heterotrimeric G-proteins, as well as Src, undergo lipid modification facilitating their association with cholesterol-rich membrane domains.

Interestingly, CXCR4 was found to form monomers, dimers and nanoclusters on T cells that own distinct lateral mobility characteristics (Martínez-Muñoz et al., 2018). Ligand binding was further shown to modulate CXCR4 dynamics leading to enhanced nanoclustering of the receptor that is controlled by the cortical actin, which in turn correlated with the strength of CXCR4 signaling. Consequently, cells expressing CXCR4 mutants with deficits in nanocluster formation showed impaired chemokine-driven signaling and leukocyte migration, both *in vitro* and *in vivo* (Martínez-Muñoz et al., 2018). Although not formally shown in this study, it is tempting to speculate that cholesterol molecules, by modulating the stiffness of membranes, are involved in controlling the lateral mobility of CXCR4 and the formation of nanoclusters.

Beside the above described role of plasma membrane cholesterol, altered extracellular cholesterol levels are observed under certain pathological conditions. High cholesterol levels in the blood (hypercholesterolemia) is a common risk factor for coronary heart diseases (Tall and Yvan-Charvet, 2015). Deposition of cholesterol in the subendothelial layer is effectively narrowing and hardening arteries leading to atherosclerosis. Importantly, cholesterol accumulation in atherosclerotic plaques gives rise to the formation of cholesterol crystals, which induce complement-dependent inflammasome activation (Samstad et al., 2014) and production of inflammatory chemokines (CCL2, CCL3, and CCL5), which results in leukocyte recruitment and a CCR2-driven chronic inflammatory disorder (Boring et al., 1998). Moreover, in a mouse model for atherosclerosis, namely in apolipoprotein E (ApoE)-deficient mice, cholesterol deposits and local dermal inflammation were observed to coincide with skin resident dendritic cells possessing a systemically reduced migratory behavior (Angeli et al., 2004). As dendritic cell emigration from the skin relies on CCR7-guided migration, it is tempting to speculate that extracellular cholesterol is taken up by dendritic cells and integrated into the plasma membrane where it interferes with CCR7 oligomerization and signaling. Pre-clinical studies using statins, which inhibit the HMG-CoA reductase to block cholesterol *de novo* synthesis, in ApoE-deficient mice revealed a marked regression of atherosclerosis through a CCR7-dependent emigration of foam cells from plaques (Feig et al., 2011) supporting this hypothesis.

## Role of Flotillins in Leukocyte Migration

The ubiquitously conserved membrane organizing proteins flotillin-1 (flot1), also known as reggie-2, and flotillin-2 (flot2)/reggie-1 have been reported as important regulators of leukocyte activities (Giri et al., 2007; Langhorst et al., 2007; Otto and Nichols, 2011; Guillaume et al., 2013; Bodin et al., 2014). The reggie proteins were originally described to be upregulated on goldfish retinal ganglion cells after nerve injury and subsequent axon regeneration (Schulte et al., 1997). Simultaneously, flotillins were identified as lipid raft proteins of detergent-resistant membrane fractions of marine lung tissue that float in sucrose density gradients (Bickel et al., 1997). Flotillins are also expressed at different levels in many leukocyte subsets, including neutrophils, monocytes, T cells and dendritic cells (Table 1). Both flot1 and flot2 possess N-terminal fatty acid modifications close to the prohibitin homology (PHB) domain (Figure 2) that allow their association with the plasma membrane and a direct interaction with F-actin (Langhorst et al., 2007; Guillaume et al., 2013; Bodin et al., 2014). Particularly, flotillins pile in actin-driven mobile membranes, such as lamellipodia and ruffles (Guillaume et al., 2013). The C-terminal part of flotillins contain an α-helical region required for their hetero-oligomerization, stabilization and lipid rafts association (Langhorst et al., 2007; Guillaume et al., 2013; Bodin et al., 2014). As flotillins are involved in cell-cell contacts (Guillaume et al., 2013; Bodin et al., 2014) and are able to interact with the actomyosin cytoskeleton of leukocytes (Ludwig et al., 2010), flotillins are predestinated to contribute to cell adhesion and migration processes. In neutrophils, both flotillins were found to interact with the adhesion molecule P-selectin glycoprotein ligand 1 (PSGL-1; Rossy et al., 2009). Upon chemokine stimulation, neutrophils polarized and flotillins together with other lipid raft associated signaling molecules (i.e., CD43 and ezrin/radixin/moesin proteins) accumulated at the cell’s uropod. Notably, the redistribution of flotillins preceded the one of CD43 and the ezrin/radixin/moesin proteins and required the integrity of the actin cytoskeleton, but not actin-myosin contraction (Rossy et al., 2009), suggesting that flotillins actively participate in neutrophil polarization. Spurred by these observations, Ludwig and colleagues found that flot1-deficient neutrophils and monocytes failed to efficiently migrate to inflammatory sites *in vivo* (Ludwig et al., 2010). *Ex vivo* analysis revealed that uropod formation and myosin IIa activity are compromised in flot1-deficient neutrophils (Ludwig et al., 2010).

**TABLE 1:** Expression level of scaffold proteins in various leukocyte subsets.

Scaffold protein		Leukocyte subset	Expression level*	References
flotillin	flot1	neutrophils	+++	Ludwig et al., 2010
		monocytes	++	Ludwig et al., 2010
		T cells	++	Ficht et al., 2019
		dendritic cells	++	–
	flot2	neutrophils	+++	–
		monocytes	++	–
		T cells	++	–
		dendritic cells	++	–
caveolin	cav1	dendritic cells	++	Oyarce et al., 2017
tetraspanin	CD9	monocytes	+++	Schenk et al., 2013
		dendritic cells	+++	Rocha-Perugini et al., 2017
	CD81	monocytes	++	Dijkstra et al., 2008
		dendritic cells	++	Mantegazza et al., 2004
	CD63	dendritic cells	++	Mantegazza et al., 2004
	CD82	dendritic cells	+++	Mantegazza et al., 2004
	CD37	dendritic cells	+++	Jones et al., 2016
				Gartlan et al., 2013
	CD151	T cells	++	Zelman-Toister et al., 2016

*Expression level based on ImmGen Microarray database (++>80; +++>800)

In T cells, flotillins also accumulate at the uropod upon exposure to chemotactic signals. Moreover, flotillins in these cells were shown to bind to actin and to regulate the actin cytoskeleton (Langhorst et al., 2007; Neumann-Giesen et al., 2007), suggesting that flotillins are required for optimal T cell migration. Recently, Ficht and colleagues demonstrated that migrating CD8^+^ T cells retrieved from flot1-deficient mice indeed displayed significant altered shape changes and motility *in vitro* and *in vivo* (Ficht et al., 2019). Surprisingly, CD8^+^ T cell homing to lymphoid organs was comparable in wild-type and flot1-deficient mice (Ficht et al., 2019).

**Box 1.**

Role of tetraspanins in antigen presentation. Orchestrated leukocyte migration is essential to launch innate and adaptive immune responses. Homing of dendritic cells to draining lymph nodes and the presentation of peripherally acquired antigens derived from pathogens to T cells conjointly dictate the quality of an adaptive immune response. Importantly, T cells migrate within lymph nodes in search for cognate antigens presented by dendritic cells. Hence the role of tetraspanins in leukocyte migration must also be discussed in the light of antigen presentation by major histocompatibility complex (MHC) molecules. Notably, many tetraspanins expressed by dendritic cells not only influence their ability to migrate but also influence antigen presentation. In fact, CD9, CD53, CD81, CD151, and CD37 were shown to associate with MHCII molecules on the cell surface of dendritic cells to augment antigen presentation (Angelisová et al., 1994; Szöllósi et al., 1996; Engering and Pieters, 2001; Saiz et al., 2018). Other members of the tetraspanin family, namely, CD63 and CD82, regulate antigen processing, MHCII biosynthesis and/or transport to the cell surface (Hammond et al., 1998; Mantegazza et al., 2004; Untemaehrer et al., 2007; Saiz et al., 2018). Several members of the tetraspanin family (CD37, CD53, CD63, CD81, and CD82) are expressed by human antigen presenting cells (Escola et al., 1998; Hammond et al., 1998; Van Den Hoorn et al., 2012) and have therefore been proposed as potential target candidates for treating inflammation and immune-mediated chronic diseases (Jin et al., 2018). More information on the role of tetraspanins in antigen presentation can be found in a recent review by Saiz et al. (2018).

**Box 2.**

Role of scaffold proteins and cancer. Cancer progression and metastasis formation are clearly linked to migration. Although not discussed in this review, it is important to note that the expression of the three families of scaffold proteins discussed in this review are implicated in cancer. Enhanced expression of flot2 was detected in samples of breast cancer and mice lacking flot2 expression showed a significantly reduced tumorigenicity and metastatic capability (Berger et al., 2013). This finding is in line with other studies that proposed the presence of flotillins as a marker for poor prognosis in breast cancer (Banning et al., 2014; Ou et al., 2017), melanoma (Liu et al., 2015), and gastric cancer (Zhu et al., 2013). Similarly, high expression of the tetraspanin CD151 has been proposed as a maker for poor prognosis in a number of metastatic tumors (Franco et al., 2010; Voss et al., 2011; Deng et al., 2012; Kwon et al., 2012; Lee et al., 2013; Li et al., 2013; Sachs et al., 2014; Yu et al., 2018; Jiang et al., 2019). The role of CD9 in cancer remains controversial and seems to vary among different cancer types. Despite promising results obtained in pre-clinical mouse models (Beckwith et al., 2015), CD37 is the only targeted tetraspanin that has moved to clinical studies (de Winde et al., 2017). CD37 is highly expressed in malignant B cells, but not on solid tumors, which makes it suitable for immunotherapy (de Winde et al., 2017). Reduced or absent expression of cav-1 strongly correlated with a poor prognosis in cancer patients. This was attributed to altered signaling in tumor cells and changes in the metabolic tumor environment as reviewed elsewhere (Martinez-Outschoorn et al., 2015).

In conclusion, flot1 emerges to play a critical role in myeloid cell migration by facilitating cell polarization, whereas in CD8^+^ T cell migration flot1 plays an unexpectedly minor role. The contribution of flot2, i.e., using flot2-deficient mice, in leukocyte migration has not been studied yet. Further studies are hence mandatory to decipher the precise role of the two flotillin proteins in the migratory behavior of different leukocyte subsets.

## Role of Caveolin-1 in Dendritic Cell Migration

The cav family constitutes of three isoforms, namely cav-1, cav-2, and cav-3, of which cav-1 is best characterized. The two splicing variants of cav-1, cav-1α, and cav-1β (Okamoto et al., 1998), not only localizes at the plasma membrane, but also at endomembranes, such as the ER, the Golgi, endosomes, and mitochondria, as well as at lipid droplets (Parton and Howes, 2010). Cav-1 is constituted of an N-terminal domain, followed by a scaffold domain, an integral membrane domain and a C-terminal domain (Root et al., 2015). The integral membrane domain includes two α-helices, which are connected by a linker region forming a U-shaped conformation that penetrates deep into the lipid bilayer of the membrane (Rui et al., 2014). Major post-translational modifications, including phosphorylations at the N-terminal domain (on tyrosine14 and serine80) and palmitoylations on three cysteine residues located at the C-terminal domain (Krishna and Sengupta, 2019), not only anchor cav-1 in the membrane but also facilitates cav oligomerization and cholesterol transport (Monier et al., 1996; Okamoto et al., 1998). Although cav proteins are predominately expressed in epithelial cells, endothelial cells, fibroblasts, and adipocytes, they are also present in leukocytes (Harris et al., 2002; Tomassian et al., 2011; Table 1). Importantly, cav-1 was reported to be upregulated in dendritic cells upon exposure to pathogen-derived danger signals (Oyarce et al., 2017). Pathogen encountering also provokes the induction of CCR7 and subsequent migration of dendritic cells to the draining lymph where the dendritic cells present pathogen-derived antigens to T cells to launch an adaptive immune response (Hauser and Legler, 2016). Notably, cav-1-deficient dendritic cells migrate significantly less towards the CCR7 chemokine ligand CCL21 compared to cav-1 proficient cells (Oyarce et al., 2017). Interestingly, the intrinsic random cell motility was not affected in dendritic cells lacking cav-1 (Oyarce et al., 2017), suggesting that cav-1 contributes to directional cell locomotion. Mechanistically, danger signal challenged dendritic cells retrieved from wild-type mice possessed significantly more actin-rich protrusions and filopodia than cav-1-deficient cells. In addition, CCR7-driven activation of the GTPases Rac1, known to promote actin protrusions, was impaired in dendritic cells lacking cav-1 (Oyarce et al., 2017). Collectively, this study suggests that cav-1 control chemokine-mediated Rac1 activation, cytoskeleton rearrangement and migration of dendritic cells *in vitro* and *in vivo*.

## Role of Tetraspanins in Leukocyte Migration

The family of tetraspanins, also known as the transmembrane 4 superfamily (TM4SF), comprises 34 members in mammals that are highly conserved among species (Adell et al., 2004; Huang et al., 2005). Tetraspanins are composed of four transmembrane domains, a small and large extracellular loop (termed SEL and LEL, respectively), and two intracellular tail domains (Seigneuret et al., 2001; Levy and Shoham, 2005; Figure 2). The LEL domain accounts for most interactions with environmental stimuli, while the cytoplasmic regions are linked to cytoskeletal and signaling molecules. The four transmembrane domains are quite flexible and facilitates the formation of the so-called tetraspanin webs or tetraspanin-enriched microdomains (TEMs) by neighboring tetraspanins (Kitadokoro et al., 2001; Hemler, 2005; Levy and Shoham, 2005; Seigneuret, 2006). Generally, tetraspanin webs act as important signaling platforms that control signaling, cell invasion, cell–cell fusion, cell adhesion, antigen presentation (Box 1), as well as cell migration.

Members of the tetraspanin family relevant for leukocyte migration, including information on the expression level, are listed in Table 1. In T cells, the tetraspanin CD151 was shown to form complexes with integrins (VLA-4 and LFA-1), and its activation was found to augment chemokine-mediated actin polymerization and migration *in vitro* (Zelman-Toister et al., 2016). Monocytes express the tetraspanins CD9 and CD81, and their cross-linking by specific antibodies was shown to significantly improve their ability to migrate across endothelial monolayers *in vitro* (Dijkstra et al., 2008; Schenk et al., 2013). Immature dendritic cells were shown to express the tetraspanins CD9, CD63, CD81, CD82, and CD151, of which CD9 and CD81 are mostly expressed at the cell surface, whereas CD63, CD82, and CD151 also localize in intracellular organelles (Mantegazza et al., 2004). Antibody-mediated cross-linking of CD9, CD63, CD81, and CD82 substantially enhanced immature dendritic cell migration *in vitro* towards the inflammatory chemokines CCL3 and CCL15, while cross-linking CD151 showed no effect (Mantegazza et al., 2004). Interestingly, cross-linking CD81 on mature, danger signal challenged dendritic cells inhibited their *in vitro* migration abilities towards the lymph-node homing chemokine CCL21 (Nattermann et al., 2006). Using gene-targeted mice revealed that CD9-deficient dendritic cells migrated readily towards CCL21 *in vitro* and migrated from the skin to inguinal lymph nodes *in vivo* (Rocha-Perugini et al., 2017). Collectively, these studies provide evidence that the tetraspanin CD9 contributes to, but is dispensable for dendritic cell migration. By contrast, migration of dendritic cells from the skin to draining lymph nodes in a contact sensitization model (FITC skin painting) was impaired in mice lacking CD37 (Gartlan et al., 2013), while mice lacking CD82 display the opposite phenotype (Jones et al., 2016). Both CD37 and CD82-deficient dendritic cells lack cellular projections. Nevertheless, CD37^–/–^ dendritic cells poorly spread under low shear flow conditions on fibronectin, while CD82^–/–^ dendritic cells showed increased cell spreading (Jones et al., 2016). Interestingly, immature dendritic cells, defined as CD37^*hi*^CD82^*lo*^ (as found in the skin), are highly motile cells owing a limited ability to activate naive T cells, while matured dendritic cells, defined as CD37^*lo*^CD82^*hi*^ (which have been exposed to pathogens and homed to lymph nodes), are less motile but show a well-orchestrated antigen presentation machinery to efficiently activate naive T cells (Jones et al., 2016). These observations strengthen the notion that leukocyte migration, cell–cell interaction and antigen presentation are interconnected processes than conjointly regulate immunity (see Box 1).

Mechanistic insights into how tetraspanins regulate cell migration are sparse. However, dendritic cells and neutrophils lacking CD37 have deficits in actin polymerization, cell spreading and polarization, which can partially be attributed to deregulated Rac1 activation and accelerated β_2_-integrin internalization, which conjointly result in impaired cell adhesion (Jones et al., 2016). Generally speaking, the family of tetraspanins play versatile roles in modulating various leukocyte functions, including migration. More to that, a single member of the tetraspanin family fulfills distinct functions that depend on subcellular localization, the differentiation stage of the cell, as well as on the environmental context the cell is navigating through.

## Conclusion and Outlook

Accumulated evidence underpins the critical role of cellular cholesterol in regulating chemokine receptor signaling and functions. Particularly, the presence of cholesterol is essential for chemokine receptor stability, ligand binding, and hence receptor function. Moreover, recent advances indicate that pathogen-derived danger signals modulate cholesterol levels in dendritic cells, which in turn affects their migratory capacities. Similarly chemokine receptor nanocluster, which is presumably regulated by cholesterol, emerges to control the signaling strength and consequently lymphocyte migration. The three membrane scaffold protein families have in common that they contribute to the formation, organization and maintenance of specialized membrane compartments. Flotillins redistribute in migrating leukocytes. Notably, while chemokine-driven cell polarization and spatio-temporal redistribution of flotillins are observed in myeloid cells and lymphocytes, flotillins are fundamentally required for directional myeloid cell migration, but seem to be dispensable for T cell migration *in vivo*. This suggests that different leukocyte subsets possess alternative adaptation modes for efficient cell migration. Caveolin-1 controls chemokine-driven Rac1 activation to promote cytoskeleton rearrangements and migration of dendritic cells *in vitro* and *in vivo*. Several tetraspanins, CD37 and CD82 in particular, play a role in regulating leukocyte migration although the molecular mechanism(s) are far from being fully understood. Further studies are required for a more comprehensive understanding of how membrane compartmentalization and membrane scaffold proteins control cell migration in general. This becomes evident by the fact that these scaffold proteins also affect cancer cell migration and metastasis formation as briefly summarized in Box 2. In general, new knowledge will be key to understand how membrane compartments in concert with membrane-associated or – spanning proteins orchestrated cell migration in health and disease.

## Author Contributions

GS prepared the figures. Both authors wrote the manuscript.

## Conflict of Interest

The authors declare that the research was conducted in the absence of any commercial or financial relationships that could be construed as a potential conflict of interest.
